# Molecular imaging of brain localization of liposomes in mice using MALDI mass spectrometry

**DOI:** 10.1038/srep33791

**Published:** 2016-09-21

**Authors:** Annabelle Fülöp, Denis A. Sammour, Katrin Erich, Johanna von Gerichten, Peter van Hoogevest, Roger Sandhoff, Carsten Hopf

**Affiliations:** 1 Center for Applied Research in Applied Biomedical Mass Spectrometry (ABIMAS). Paul-Wittsack-Str. 10, 68163 Mannheim, Germany; 2 Instrumental Analytics and Bioanalytics, Mannheim University of Applied Sciences, Paul-Wittsack-Str. 10, 68163 Mannheim, Germany; 3 Lipid Pathobiochemistry, German Cancer Research Center (DKFZ), Im Neuenheimer Feld 280, 69120 Heidelberg, Germany; 4 Phospholipid Research Center, Im Neuenheimer Feld 515, 69120 Heidelberg, Germany; 5 Institute of Medical Technology, University of Heidelberg and Mannheim University of Applied Sciences, Paul-Wittsack-Str. 10, 68163 Mannheim, Germany

## Abstract

Phospholipids have excellent biocompatibility and are therefore often used as main components of liposomal drug carriers. In traditional bioanalytics, the *in-vivo* distribution of liposomal drug carriers is assessed using radiolabeled liposomal constituents. This study presents matrix-assisted laser desorption/ionization mass spectrometry imaging (MALDI MSI) as an alternative, label-free method for *ex-vivo* molecular imaging of liposomal drug carriers in mouse tissue. To this end, indocyanine green as cargo and two liposomal markers, 1,2-dipalmitoyl-*sn*-glycero-3-phosphoglycerol (DPPG) and 1,2-distearoyl-*sn*-glycero-3-phosphoethanolamine conjugated with monodisperse polyethylene glycol (PEG_36_-DSPE) were incorporated into liposomal carriers and administered to mice. We used MALDI MSI of the two lipid markers in both positive and negative ion mode for visualization of liposome integrity and distribution in mouse organs. Additional MSI of hemoglobin in the same tissue slice and pixel-by-pixel computational analysis of co-occurrence of lipid markers and hemoglobin served as indicator of liposome localization either in parenchyma or in blood vessels. Our proof-of-concept study suggests that liposomal components and indocyanine green distributed into all investigated organs.

Phospholipids are main components of cellular membranes and therefore have excellent biocompatibility. This makes them most appropriate as important pharmaceutical excipients and main components of liposomal drug carriers. Conventional first generation liposomes contain mixtures of neutral phospholipids like egg phosphatidylcholine (EPC) and cholesterol^1^,^2^. The latter increases the stability of liposomes in the presence of plasma^3^. However, rapid uptake by cells of the reticuloendothelial system very quickly removes these liposomes from circulation^4^. Second generation long circulating liposomes, so-called ‘stealth liposomes’, include the synthetic polymer polyethylene glycol (PEG)^5^,^6^,^7^. For this approach, lipids with PEG-functionalized head groups are incorporated at a low percentage (5–10%) of total lipids into liposomes, and the polymer part is generally exposed on their outside surface. PEGylated carriers extend blood-circulation time while reducing uptake by the mononuclear phagocyte system. Prolonged circulation with PEG is independent of cholesterol content, degree of saturation in either the phosphatidylcholine or the phosphatidylethanolamine lipid anchor, lipid dose, or addition of other negatively charged lipids like phosphatidylglycerol^8^. Today, both conventional- and stealth-liposomes have entered the research mainstream of drug-delivery systems for the *in-vivo* delivery of multiple cargos from small molecule therapeutics to nucleic acids^2^,^9^. So have been Doxil and Myocet the first approved liposome-based drugs for cancer treatment. Nevertheless, evaluation of distribution and drug release of pharmaceutical liposomes *in-vivo* remains a major challenge during development of liposomal drugs. Magnetic resonance imaging (MRI) can assist in both localizing drug carriers and monitoring the release of their content^10^,^11^. However, MRI offers limited spatial resolution (e.g. 200 × 200 × 1000 μm^3^ with good contrast) and its use requires the incorporation of specific radioactive labels. In contrast, label-free methods for non-invasive *in-vivo* or *ex-vivo* measurements of drug carrier distribution at therapeutic concentrations have not been reported yet.

Matrix-assisted laser desorption/ionization time-of-flight mass spectrometry imaging (MALDI MSI)^12^,^13^ can be used to determine the spatial distribution of various compounds in tissue with a single label-free measurement. MALDI MSI has been used for a variety of applications, ranging from bioanalysis^14^,^15^ of lipids^16^,^17^, proteins, peptides, drugs and their metabolites^18^ to the detection of polymers in a biological environment^19^,^20^. Although several imaging applications for the visualization of abundant, endogenous phospholipid species exist^16^, MALDI MSI of phospholipids in phospholipid-based liposomal drug carriers *in-vivo* has not been reported yet. Analytical method development for applications of MALDI MSI in the drug delivery field are generally underexplored, as only a single study monitoring the non-natural cationic lipid DLin-KC2-DMA in lipid nanoparticle-formulated siRNA by MALDI MSI in mice has been published^21^.

Here, we present a MALDI MSI method for imaging of liposomal drug carriers in the presence of a matrix of natural endogenous phospholipids. To this end, we imaged three liposomal components after *in-vivo* dosing of mice using MALDI mass spectrometry. The results suggest possible utility of MALDI MSI as a non-radioactive tool for formulation and drug delivery studies.

## Results

### Design of liposomal carriers and strategy for monitoring of liposomal integrity

Radiolabeled liposomal constituents enable evaluation of the *in-vivo* distribution of liposomal drug carriers with very high sensitivity. However, since only a single radiolabel can be monitored at any given time, radiochemical methods do not permit the evaluation of *in-vivo* liposome integrity or cargo retention and release. We hypothesized that MALDI MSI as a label-free method for simultaneous imaging of multiple masses may enable an innovative workflow that combines multiple MSI experiments across different mass scales in both polarities to address these questions in a single tissue slice. The main rationales for the present study was the following: If liposomal carriers contained two distinct markers that could be visualized by MSI, then non-co-localization would indicate disintegration, whereas co-localization would indicate liposome integrity. Similarly, co-localization of the liposome marker ion signals with the one for hemoglobin would indicate that the liposome was in a blood vessel. To this end, we chose two marker lipids that served as liposomal mass markers.

**Figure 1:**
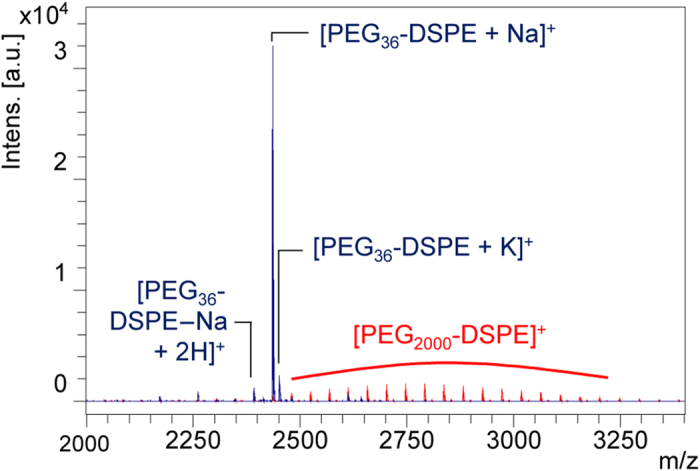
Comparison of MALDI MS spectra of PEG2000-DSPE and monodisperse PEG_36_-DSPE. MALDI spectra of 0.5 mg/mL PEG_36_-DSPE (blue) or PEG2000-DSPE (red), measured with PhCCAA matrix (5 mg/mL in acetone/water (90:10, v/v)).

Endogenous phosphatidylglycerols display nearly no distinct background signals, as only small amounts are observed in tissue besides the lung^22^. Furthermore, phosphatidylglycerols are componens of known liposomal formulations. Therefore, we added DPPG (*m/z* 721.5) as a marker to the liposomal drug carriers besides egg phosphatidylcholine (EPC) and cholesterol. Since PEG-conjugated lipids are often used in drug formulations as a means for increasing plasma half-life of liposomes, we chose a synthetic polyethylenglycol- (PEG-) conjugated lipid as the second distinct mass marker for liposomes. However, PEGs are commonly synthesized by polymerization of ethylene oxide and are commercially available as mixed polymers that cover a wide range of molecular masses. Consequently, the often-used PEG2000-1,2-distearoyl-*sn*-glycero-3-phosphoethanolamine (DSPE) shows a broad molecular mass distribution that results in dilution of signal and decreased intensity (Fig. 1). Therefore, we incorporated the monodisperse *N*-(Carbonyl-methoxypolyethylenglycol-1618)-DSPE (PEG_36_-DSPE; *m/z* 2435.5 [M+Na]^+^, *m/z* 2451.5 [M+K]^+^, *m/z* 2467.5 [M-Na+2K]^+^), a defined 36-mer, into liposomes. Finally, we incorporated indocyanine green (ICG)^23^, the first fluorophore approved to test hepatic function in the 1970s^24^. It is a well-known drug surrogate that is established in many clinical applications and has been used in humans as well as animals for cancer detection using optical imaging^25^. In conclusion, our model liposomes were prepared with EPC, DPPG, cholesterol and PEG_36_-DSPE in a molar ratio of 27.5:27.5:40:5 and were loaded with 2 mM ICG. The particle size distribution had a Z-Average of 216.2 ± 0.8 nm, as assessed by dynamic light scattering.

### PhCCAA as versatile MALDI matrix for MALDI MS of liposomal components

For DPPG imaging in negative ion mode the matrix 4-Phenyl-*α*-cyanocinnamic acid amide (PhCCAA) that we had introduced for the MALDI MS imaging of multiple lipid classes including phosphatidylglyceols^26^ was used. During the same MALDI MS imaging experiment ICG was detected. However, the ICG analysis on a steel target or spotted onto mouse liver tissue (Suppl. Figure S01) confirmed adduct formation and loss of a methyl group. Whereas the predominant signal on steel target, i.e. without ion suppression by tissue, was [ICG-H]^−^ (*m/z* 751.3) the most intense ICG signals on tissue were [ICG-CH_3_-H+Na]^−^ (*m/z* 759.3) or the [ICG-CH_3_-H+K]^−^ (*m/z* 775.2). In contrast, light-induced [2 + 2] cycloaddition of singlet oxygen and resulting decomposition of ICG^27^ was negligible during MALDI MSI experiments.

Whereas for MALDI MSI of phospholipids in positive ion mode 2,5-dihydroxybenzoic acid (DHB) matrix is often preferred^16^, the suitable matrix for the detection of synthetic polymers depends on their chemical structure^28^. Often the best spectra are obtained if solubility of polymer and matrix match^29^,^30^. Therefore, polar matrices like DHB or 4-Hydroxy-*α*-cyanocinnamic acid (HCCA) may be suitable for hydrophilic polyethylene glycol species, but no report for MALDI MSI of PEG-lipid hybrids in tissue has been published. Although DHB has successfully been used for MALDI MSI of synthetic PEG^31^, the laser threshold for DHB is high for a 355 nm Smartbeam II laser. Excess energy may cause fragmentation of PEG_36_-DSPE (Suppl. Figure S02A). Therefore, we compared three different matrices: While DHB and HCCA resulted in higher intensities for the polymer-lipid-hybrid, PhCCAA showed the lowest background on MALDI steel targets (Suppl. Figure S02B). For comparison of matrices after administration of PEG_36_-DSPE containing liposomes to mice, reported sample preparation methods for detection of polymers with DHB and HCCA matrix^20^ were compared to the sample preparation method for lipid detection with PhCCAA matrix that we reported earlier^26^ (Suppl. Figure S02C). Although DHB was the best choice for detection of polysulfone and polyvinylpyrrolidone^20^, it was not possible to detect the PEG-lipid-hybrid after administration to mice with this sample preparation method. HCCA matrix led to strong fragmentation, whereas PhCCAA showed the lowest fragmentation of PEG_36_-DSPE on the tissue compared to the starting material. Take advantage of the opportunity to detect all liposomal components with the same sample preparation method, we chose PhCCAA for detection of DPPG and ICG in reflector negative mode and PEG_36_-DSPE in reflector positive mode.

### MALDI MS imaging of liposomal drug carrier and hemoglobin for identification of the localization in mouse brain sections

**Figure 2:**
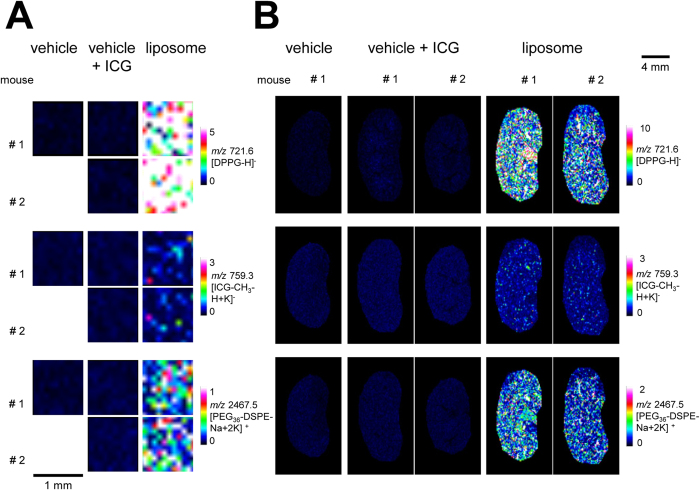
MALDI MSI on liver and kidney tissue of mice that were dosed with control or liposomes. MALDI MSI was performed on liver slices (**A**) or kidney slices (**B**) of mice that were either dosed with vehicle (PBS), vehicle + ICG (ICG in PBS) or liposomes containing the inspected analytes: DPPG, PEG_36_-DSPE and ICG. DPPG and ICG were detected in reflector negative ion mode and PEG_36_-DSPE in reflector positive mode, all with PhCCAA matrix (5 mg/mL in acetone/water (9:1, v/v)).

**Figure 3:**
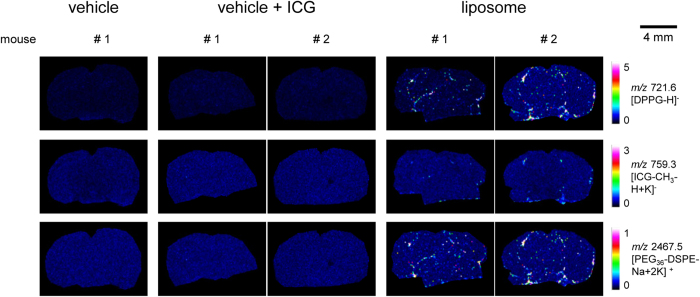
MALDI MSI on brains of mice that were dosed with vehicle or liposomes. MALDI images were generated from brain slices of mice that were either dosed with vehicle (PBS), vehicle + ICG (ICG in PBS) or liposomes containing the inspected analytes: DPPG, PEG_36_-DSPE and ICG. DPPG and ICG were detected in reflector negative ion mode and PEG_36_-DSPE in reflector positive mode. Imaging was performed with PhCCAA matrix (5 mg/mL in acetone/water (9:1, v/v)).

**Figure 4:**
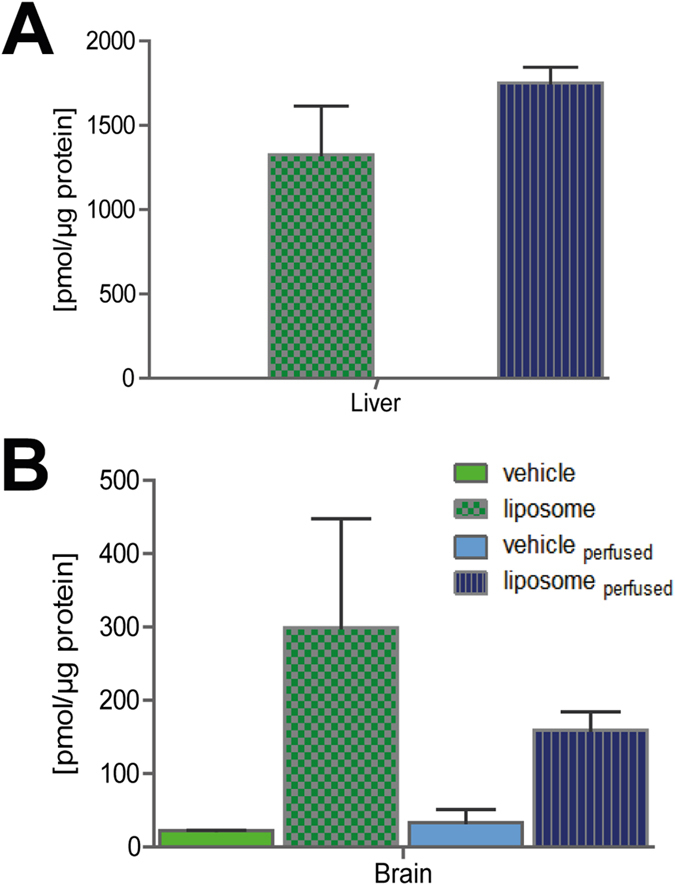
UPLC-ESI-MS/MS quantification of DPPG in mice livers and brains. UPLC-ESI-(QqQ)MS^2^ quantification of DPPG in **(A)** liver and **(B)** brain of mice that were dosed with vehicle (PBS) or liposomes containing DPPG, PEG_36_-DSPE and ICG. Half of the animals in each group were sacrificed-perfused to eliminate blood from brain vessels.

For MALDI MS imaging, ICG-loaded carriers with two marker lipids were administered to mice (DPPG: 205 mg/kg; ICG: 8.5 mg/kg; PEG_36_-DSPE: 120 mg/kg), and images of dosed animals were compared with those of control animals dosed with PBS (vehicle) or ICG in PBS (vehicle + ICG; ICG: 8.5 mg/kg). Whereas DPPG and PEG_36_-DSPE showed intense signals during MALDI MS imaging measurements of liver (Fig. 2A) or kidney (Fig. 2B) of liposome-dosed mice, ICG was barely detectable, presumably because of its lower dose and dilution of signal onto several masses. In contrast to peripheral organs, transport of drugs and drug carriers from the circulating blood into the brain is restricted by the blood-brain-barrier (BBB). The liposomes we designed did not contain any brain-targeting modifications such as cationized albumin, transferrin or apolipoprotein E fragements^7^. Surprisingly though, liposomes were detected in the brain of dosed mice (Fig. 3). We therefore suspected that the measured signals represented liposomes in the circulating blood and that identification of non-targeted liposomal drug carriers used in this study outside of blood vessels would be a rare event at best. For verification of the localization of liposomes in brain, we sought to remove blood. Therefore, half of the experimental animals were perfused^32^ before being sacrificed. Following perfusion, reduced ICG-fluorescence in brains of liposome-dosed mice (Suppl. Figure 04A) indicated the successful reduction of blood in that tissue. However, residual ICG fluorescence suggested that either blood removal was incomplete or that ICG had entered brain tissue. For further verification of the presence of liposomes in tissues of dosed animals, we subjected brain and liver homogenates to UPLC-ESI-(QqQ)MS^2^ analysis. Phosphatidylglycerols including DPPG were detected as protonated and ammoniated molecular ions in positive ion mode during ESI-MS^2^ measurement (Suppl. Table 1). Intensities were summed up and normalized against the deuterated internal phosphatidylglycerol standard (PG(16:0;D_31_-18:1)). No endogenous DPPG was detected in liver extracts, and endogenous DPPG levels were negligible at <25 pmol/μg protein in brain tissue extracts (Fig. 4). In contrast, quantification of phosphatidylglycerols by LC-ESI-MS^2^ confirmed the presence of >150 pmol DPPG per μg protein in perfused brains and even higher concentrations in non-perfused brains, in which blood amount was not reduced (Fig. 4B).

**Figure 5:**
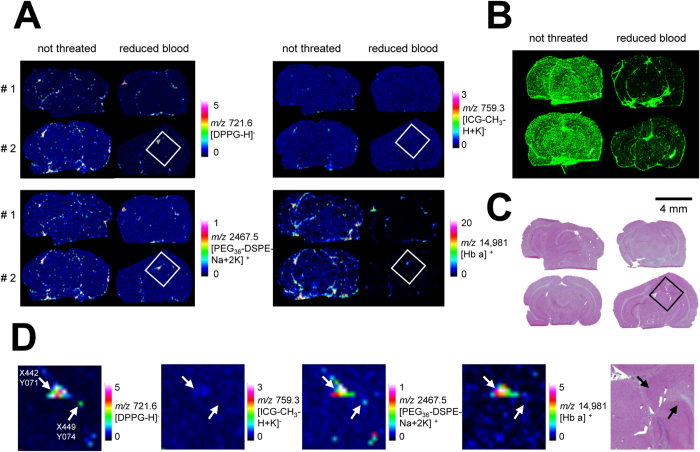
Images on brains of mice that were dosed with liposomes and localization of the detected liposomes. (**A**) MALDI MSI images were performed on brain slices of mice that were dosed with liposomes. Half of the mice were perfused before being sacrificed (right panels) to reduce the remaining blood in the tissue. MALDI images of DPPG and ICG were acquired in reflector negative ion mode, of PEG_36_-DSPE in reflector positive mode and of HB *α* chain in linear positive mode. DPPG, ICG and PEG_36_-DSPE were measured with PhCCAA MALDI matrix. HB was detected after delipidation and sDHB deposition on the same tissue region. **(B)** Fluorescence images performed on brain slices of mice that were dosed with liposomes. **(C)** HE staining of tissue slices measured with MALDI MSI. **(D)** Magnification of MALDI MS images of the boxed parts marked in perfused brain in (**A**) and HE stained brain in (**C**). Pixels indicated by an arrow shows the co-localisation of the liposomal components and hemoglobin at pixel X442 Y071 and the absence of HB at pixel X449 Y074.

An advantage of MALDI MSI is the possibility to perform multiple measurements with different settings on the same tissue, in order to detect a broad range of biomolecules^33^. For instance, simultaneous and correlative MS imaging of drug and heme group has been validated as a method to visualize the lumen of the blood vessels in the brain and, thus, permeability through the BBB^34^. Therefore, we examined the presence of remaining blood in the same tissue slice in a simple label-free way by MALDI imaging of hemoglobin (HB). We imaged first DPPG and ICG in negative ion reflector mode and PEG_36_-DSPE in positive ion reflector mode with PhCCAA matrix and afterwards HB in positive ion linear mode with sDHB matrix (Fig. 5A). Prior to sDHB application and protein imaging, the tissue was washed to remove PhCCAA and the lipid background. The imaged signals of liposomal components showed lower intensity in single spectra measured on the tissue of perfused mice. But DPPG, PEG_36_-DSPE, ICG (weak) as well as low amounts of HB were detected even in the brains of perfused mice by MALDI MSI. To conduct in-depth computational evaluation of these MS images, we programmed an analysis tool in R for pixel-by-pixel correlation analysis of the three *m/z* values for DPPG, PEG_36_-DSPE and HB. In two brain slices from non-perfused mice, 57% and 72% of all pixels contained HB, whereas only 11% of pixels in both perfused brain slices contained HB.

We therefore performed computational correlation analysis on slices from perfused brains: As expected, almost all pixels that displayed signals for the two lipid markers also provided analytical evidence for the blood marker HB suggesting that liposomes were present in blood vessels of the brain. A single pixel spectrum measured at motor position X442 Y071 served as an example for this notion (Suppl. Figure 05A): Besides the signals of DPPG, ICG (A1) and PEG_36_-DSPE (A2), the signal of HB (A3) was also evident for this pixel. Neighboring single pixel spectra showed the same signals. Therefore, it is highly likely that the imaged structure shown in the magnification (Fig. 5B) represents a blood vessel that was not fully rinsed by perfusion. Apparently, residual liposome-containing blood remained after the perfusion procedure. In contrast, in pixel X449 Y074 near to this presumed vessel the liposomal components DPPG and PEG_36_-DSPE but virtually no HB was detected (Suppl. Figure 05B). The apparent absence of HB from the X449 Y074 single pixel spectra and mass spectra of surrounding pixels either indicates the entrance of liposomal components into brain tissue or the presence of liposomes in a vessel where blood had been completely removed. Overall, only three such pixels (out of about 4000) were found in brains of perfused mice. In brains of non-perfused mice, no such pixels were identified. Suppl. Figure 05 shows the single spectra for all these pixels (B–D). While the pixels identified at motor position X449 Y074 (B) and X451 Y083 (C) were located in the hippocampal formation in the brain of the first perfused mouse, the pixel X494 Y153 (D) was identified in the second perfused mouse brain in the lateral forebrain bundle.

## Discussion

Formulation science and preclinical drug metabolism and pharmacokinetics (DMPK) studies would greatly benefit from non-radioactive methods for visualization of drugs and formulations in tissue. In particular, visualization of locations of intact liposomal drug carriers and monitoring of release of their content would be desired. Although MRI and fluorescence imaging are possible techniques for this purpose, their use requires contrast agents such as iron oxide nanoparticles or gadolinium chelate^10^ or the incorporation of specific radioactive or fluorescent labels that typically alter molecular properties of the vesicles^11^. Only Raman imaging^35^ and optical microscopy may provide the option to visualize liposomes without changing their composition. Successful translation from liposomal *in-vitro* visualization to *ex-vivo* imaging in tissue after dosing of liposomal drug carriers to animals has not been attempted yet. Towards this goal, new bioanalytical concepts are required to foster future technology development. Despite a growing number of applications in other fields, MALDI MSI has never been systematically studied for imaging of drug carriers. Therefore, we used MALDI MSI in a proof-of-concept study as a label-free method for simultaneous visualization of drugs and formulations in animal tissue.

Presence of markers that are absent or not abundant in the tissue of interest but indicate liposome position instead are requirements for detection of transport of liposomal drug carriers into organs by MALDI MSI. We reasoned that a single marker would not allow for unambiguous visualization of intact liposomes, since it could leak out or transfer to other lipid domains/blood components when liposomes disintegrate. Therefore, we integrated two chemically different markers into liposomes that could be visualized by MSI. We validated a set of such markers that are derived from or similar to constituents that are frequently used in drug formulations containing phospholipids. Phosphatidylglycerols are used for liposomal carrier design and can be used as marker lipids, since they display few background signals, as only small amounts are observed in tissue besides the lung^22^. For instance, all approved DepoFoam^TM^-based liposomes^36^,^37^ or Lipoplatin^38^, an alternative liposomal formulation of cisplatin in clinical studies, contain the lipid DPPG that we used for this study. Furthermore, we incorporated the monodisperse PEG_36_-DSPE that is similar to constituents used in marketed pharmaceutical liposome products and ICG to support MALDI MS images with fluorescence imaging data. ICG, the first fluorophore approved to test hepatic function in the 1970s^24^, has been used in humans as well as animals for cancer detection using optical imaging^25^ and was used in liposomes for diagnosis and therapeutic monitoring of cerebral malaria^39^. Based on this, we established a method for detecting DPPG and ICG in negative ion mode and PEG_36_-DSPE in positive ion mode using the same MALDI matrix, PhCCAA.

Using MALDI MSI, we detected all liposomal compounds in livers (Fig. 2A), kidneys (Fig. 2B) and even in brains (Fig. 3) of mice dosed with liposomes. Transport of drugs and drug carriers from the circulating blood into the brain is restricted by the BBB. Conventional liposomes do not cross it^40^,^41^,^42^. Also PEGylated liposomes like the ones used here do not cross the intact BBB^43^,^44^. Improved drug delivery across the intact BBB can be achieved by actively transported targeted drug carriers smaller than 180 nm^7^,^45^,^46^. In this study, following analysis of all liposomal components, we visualized residual blood in the same tissue slice by MALDI MSI to determine localization of liposomes either in brain parenchyma or the circulating blood. Traditional histomorphology visualizes the brain’s vascular system by dye perfusion or by immunohistochemistry (IHC) of marker proteins, both of which require specific labeling reagents^47^,^48^. In contrast, MALDI MS Imaging of heme is a label-free method for vasculature imaging that has been validated by Liu *et al.*^34^. However, since the heme group is a component of various proteins^49^, we deemed it even more specific to image *m/z* values of hemoglobin (HB) chains. Murine vessels in the 100–200 μm range can be visualized by MALDI MSI using *m/z* 14,981 (*α* chain) and *m/z* 15,617 (*β* chain)^50^ for HB^51^. Nevertheless, besides lower spatial resolution compared to other imaging methods, MALDI MSI exhibits currently lower sensitivity that may be caused by ion suppression^52^. Therefore, we used a second approach for verification of liposome localization, namely by reducing blood signals by sacrificing-perfusing half of the experimental animals. Comparing MS images of liposomal components and HB in perfused and non-perfused brains, we found the majority of liposomal components to co-localize with HB. Only three spectra of single pixels were identified by computational analysis of brains of both perfused mice where no co-localization with HB was observed. This indicated either the entrance of liposomal components into brain parenchyma or the presence of liposomes in a blood-depleted blood vessel. Absence of any single pixel spectra containing peaks for two liposomal components but no HB in non-perfused brains strongly suggests that, in accordance with the literature, the untargeted liposomes used in this study did not pass the BBB.

Our main conceptual idea for the present study was the following: If liposomal carriers contained two distinct markers, then computational assessment of co-localization (here defined as co-occurrence of the two lipid markers or of lipid markers and drug in a given pixel) could be used as a surrogate read-out for monitoring liposomal integrity and cargo retention in tissue. Non-co-localization of both markers as assessed by MALDI MSI could indicate liposomal disruption, whereas co-localization could suggest liposome integrity. It is important to note that MALDI MSI at maximum sensitivity required for this application currently precluded the use of spatial resolution lower than 50–100 μm with available MALDI-TOF MS. Hence, single-pixel MS spectra presently represent relatively large areas containing multiple liposomal particles and currently do not permit unequivocal statements about liposome integrity. Nevertheless, co-localization of two lipid markers in a pixel combined with the known reduced degradation of otherwise degradable marker lipids that are present in a liposome may arguably be the best surrogate for liposome integrity accessible by label-free methods today. Similarly, co-localization of liposome marker ion signals with the ones for HB is the current best surrogate for liposome localization in a blood vessel. Fortunately, we fully expect that available advanced Fourier transformation (FT)MS technology that enables recording of long transients as well as future developments in mass spectrometry instrumentation will overcome present limitations in sensitivity at high spatial resolution and mass resolving power. Recently, newly developed MALDI-MS instrumentation such as devices with improved spatial resolution of 1 μm and high imaging speeds^53^ or with laser-induced post-ionization^54^ has been presented. They are already a step in the right direction and, together with further improvements in lipid analysis using time-of-flight secondary ion mass spectrometry (ToF-SIMS)^55^, may further support the concept for imaging of drug carriers presented here.

In conclusion, our proof-of-concept study suggests that MSI in dual ion modes with liposomes containing two distinct molecular markers could be used for refined non-radioactive DMPK and formulation studies.

## Methods

### Materials

Solvents were generally obtained from VWR (Bruchsal, Germany) in the highest available purity. The MALDI matrix sDHB was obtained from Bruker Daltonics, 4-Phenyl-*α*-cyanocinnamic acid amide (PhCCAA)^26^ from Sirius Fine Chemicals SiChem (both Bremen, Germany). Cholesterol and indocyanine green (ICG) were from Sigma Aldrich (Taufkirchen, Germany). 1,2-Dipalmitoyl-*sn*-glycero-3-phosphoglycerol (DPPG) was purchased from Avanti Polar Lipids (Alabaster, USA). Egg phosphatidylcholine (EPC) and the sodium salt of *N*-(Carbonylmethoxypolyethylenglycol-1618)-1,2-distearoyl-*sn*-glycero-3-phosphoethanolamine (PEG_36_-DSPE) was obtained from Lipoid GmbH (Ludwigshafen, Germany).

### Liposome Preparation and purification

EPC, cholesterol and PEG_36_-DSPE were dissolved in chloroform/methanol (9:1) and DPPG in chloroform/methanol/ammonium acetate buffer (400 mM) (10:10:1) to a concentration of 100 mM. ICG (5 mM) was incorporated in liposomes by dual loading. Therefore, half of the ICG amount was dissolved in chloroform/methanol (9:1) to a concentration of 25 mM, and the other half was dissolved in PBC to a concentration of 2.5 mM. Chloroform/methanol-containing solutions were mixed in a volume ratio of EPC/DPPG/cholesterol/PEG_36_-DSPE/ICG (41.25/41.25/60/7.5/3.75, v/v) and solvents were evaporated in a SpeedVac (ThermoScientific, Waltham, USA) at 40 °C. Liposomes were prepared by dual asymmetric centrifugation (DAC)^56^ in a SpeedMixer (Hauschild Engineering, Hamm, Germany). Therefore, 70 mg glass beads (ø 0.7–1 mm) and 16.2 μL PBS that was spiked with ICG were added to the dried lipid films and the first DAC run was started (3540 rpm, 30 min). Subsequently, 27 μL PBS spiked with ICG was added and subjected to another DAC run (1 min, 3540 rpm). The last step was repeated. Finally, the liposome solution was filled up to a volume of 150 μL with ICG-spiked PBS and rested for at least 30 min. Non-incorporated ICG was removed by Size Exclusion Chromatography (Sephadex G 25 medium column, GE Healthcare, Buckinghamshire, UK). Final concentration of liposomal components (EPC/DPPG/cholesterol/PEG_36_-DSPE/ICG) was 27.5/27.5/40/5/2 (mM). Encapsulation efficiency was determined by ICG quantification on a HPLC (Agilent 1100, Waldbronn, Germany) using an UV detector at a wavelength of 366 nm. For evaluation of the size distribution of the liposomes, the Z-Average and polydispersity index (PDI) of the vesicles were determined (n = 3) using dynamic light scattering in a Zetasizer Nano-596 (Malvern Instruments, Worcs, UK).

### Animal study design, drug administration, and organ and tissue preparation

Animal experiments and organ removal was conducted by a contract research organization, Heidelberg Pharma GmbH (Ladenburg, Germany). The Heidelberg Pharma GmbH animal test facility was registered by the regional licensing body (“Regierungspräsidium Karlsruhe”, file number 35 9185.64) and the specific experiment protocol was approved by the ethics committee attached to Regierungspräsidium Karlsruhe (35-9185.81/G-35/10). The institutional ethics committee (“Tierschutzausschuss”) of Heidelberg Pharma GmbH reviewed and enforced the general compliance with animal welfare of the facility.

In brief, male C57BL/6JRj mice (5 weeks, average body weight 19–20 g) were injected *i.v.* into the tail veins either with PBS (vehicle, 2 animals), PBS spiked with 2 mM ICG (vehicle + ICG, 4 animals) or ICG-containing liposome solution (liposome, 4 animals). Application volume was 10 mL/kg body weight with a liposome concentration described above. Half of the animals in each group were sacrificed-perfused to eliminate blood from brain vessels. 3 h after dosing, animals were sacrificed, organs were rapidly removed and immediately frozen in an isopentane bath on dry ice and then stored at −80 °C. Frozen tissue was sliced into 10 μm sections for MALDI MSI using a Leica CM1950 cryostat (Leica Biosystems, Nussloch, Germany) at a temperature of −15 °C. Cryosections were mounted onto indium tin oxide (ITO)-coated conductive glass slides (Bruker Daltonics) and dried for 1 h under vacuum.

### Tissue Preparation and MALDI-TOF MS Imaging

MALDI matrices were deposited onto tissue with a SunCollect MALDI Spotter (SunChrom, Friedrichsdorf, Germany). Air pressure was 2.5 bar. Distance between tissue and spray head was 25.3 mm. PhCCAA matrix (5 mg/mL in acetone/water (9/1, v/v)) was deposited in nine layers^26^. MALDI images were acquired on an UltrafleXtreme (355 nm Smartbeam laser; 2000 Hz repetition rate). DPPG images were obtained in negative ion reflector mode in the *m/z* range from 600–1800 Da with a low mass gate at 520 Da and a spatial resolution of 100 μm with 200 laser shots per position. The MS was calibrated internally using a list of theoretical masses of detected lipids. For the PEG_36_-DSPE images a shift of 50 μm in x- and y direction was adjusted and images were obtained in positive ion reflector mode in the *m/z* range from 1000 to 4000 Da with a low mass gate at 1000 Da and a spatial resolution of 100 μm with 200 laser shots per position. The MS was calibrated externally using Peptide Calibration Standard II (Bruker Daltonics). After DPPG and PEG_36_-DSPE images, PhCCAA matrix and lipid background was removed by a six step washing protocol, described elsewhere^57^ and sDHB matrix (60 mg/mL in ACN/water (2/3, (v/v) + 0.5% TFA)) was deposit in 5 layers for protein measurements (slow flow rate: 10 μL/min for the first two layers, 15 μL/min for all other layers). A drying step of 3 min was introduced between each cycling step. After replacing PhCCAA with sDHB matrix, protein acquisition was done on the same positions as for the DPPG image but in linear positive ion mode in the *m/z* range from 5–20 kDa with a low mass gate at 5 kDa and a spatial resolution of 100 μm with 500 laser shots per position. The MS instrument was calibrated externally using Protein Calibartion Standard I (Bruker Daltonics). MALDI MS imaging acquisition and data visualization was performed using flexImaging 4.1 software. Therefore, mass filters were chosen with a width of 0.2 Da for reflector and 2 Da for linear mode measurements. To avoid artefact generation, normalization was not done.

### MALDI MS Data Analysis

The resulting data set comprised three individual MS imaging spectral data cubes (DPPG/reflector negative mode, PEG_36_-DSPE/reflector positive mode and protein/linear positive mode). In it, pixel dimensions and pixel locations were matched for all three data cubes. Subsequently, each data cube was individually baseline-corrected and median-normalized in FlexImaging 4.1 (Bruker Daltonics). Mass ranges of the data sets were clipped individually to only cover the mass ranges that contain the specific mass peaks of interest before exporting them in imzML format^58^. Afterwards, the data cubes were imported into R 3.2.3 (R Foundation for Statistical Computing, Vienna, Austria)^59^ using the Cardinal Package. To be able to detect the presence of a specific mass peak within the three data sets, peak picking based on an interpolated constant noise pattern along the clipped mass ranges was performed on each spectrum. To maintain high signal intensity and avoid picking noise spikes, no smoothing was performed and high signal-to-noise ratios were chosen (S/N ≥ 10 for DPPG and PEG_36_-DSPE, S/N ≥ 15 for protein) for the peak picking algorithm. Picked signals were extracted and re-assigned to their respective pixels and data sets. Finally, an iterator was constructed which iterated simultaneously along the three data set planes to be able to quantify the instances of co-occurrence of the DGGP and PEG_36_-DSPE signal simultaneously with the absence of the hemoglobin signal at the same pixel location. To compensate for the mass spectra misalignment between the three data sets, the peak acceptance criteria were set to 721.6 Da ± *tol* for DPPG, 2467.5 Da ± *tol* for PEG_36_-DSPE, and 14981.0 Da ± *tol* for protein (*tol* is a constant equal to the average half peak width and has the value of 0.5 Da for DPPG and PEG_36_-DSPE, and 5 Da for linear mode protein data).

### Optical Images

Optical images of the stained sections were acquired by first removing the covering MALDI matrix by rinsing MSI-analyzed sections with a series of organic solvents (50% MeOH, 70% MeOH, 2 × 100% MeOH and finally acetone). Hematoxylin and eosin (H&E) stainings were performed using standard protocols, and optical images were acquired with a Biorevo BZ 9000 (Keyence) microscope.

### Fluorescence Images

The fluorescence images of ICG were detected in 10 μm thin organ section mounted on the ITO-coated conductive glass slides with the LI-COR Odyssey (21 μm resolution, 0 mm offset with highest quality). Channel sensitivity was optimized for the complete set of sections. Sensitivity setting were set to 3.5 (brain) or 1.5 (kidney and liver) for the 800 nm channel and 1.0 for the 700 nm channel.

## Additional Information

**How to cite this article**: Fülöp, A. *et al.* Molecular imaging of brain localization of liposomes in mice using MALDI mass spectrometry. *Sci. Rep.* **6**, 33791; doi: 10.1038/srep33791 (2016).

## Supplementary Material

Supplementary Information
