# Supplementary material for: Molecular imaging of brain localization of liposomes in mice using MALDI mass spectrometry
Source: Sci Rep. 2016 Sep 21;6:33791. doi: 10.1038/srep33791 (PMC5030664; doi:10.1038/srep33791)
Supplement: Supplementary Information [file srep33791-s1.pdf]

Supplementary Information

Molecular imaging of brain localization of liposomes in mice using MALDI mass spectrometry

Annabelle Fülöp [1,2], Denis A. Sammour [1,2], Katrin Erich [1,2], Johanna von Gerichten [4], Peter van Hoogevest [5], Roger Sandhoff [1,4], and Carsten Hopf [1,2,3, #]

1 Center for Applied Research in Applied Biomedical Mass Spectrometry (ABIMAS),
2 Instrumental Analytics and Bioanalytics, Mannheim University of Applied Sciences, Paul-Wittsack-Str. 10, 68163 Mannheim, Germany
3 Institute of Medical Technology, University of Heidelberg and Mannheim University of Applied Sciences, Paul-Wittsack-Str. 10, 68163 Mannheim, Germany
4 Lipid Pathobiochemistry, German Cancer Research Center (DKFZ), Im Neuenheimer Feld 280, 69120 Heidelberg, Germany
5 Phospholipid Research Center, Im Neuenheimer Feld 515, 69120 Heidelberg, Germany

[#] Correspondence should be addressed to: carsten.hopf@medtech.uni-heidelberg.de

	**Page**
Suppl. Methods: Lipid Extraction and DPPG quantification	2
Suppl. Figure S01: MALDI spectra of ICG.	3
Suppl. Figure S02: Structure of PEG_{36}-DSPE and MALDI-TOF spectra.	3
Suppl. Figure S03: Fluorescence images.	4
Suppl. Figure S04: UPLC and ESI-(QqQ)MS^2 profiles of liver and brain extracts.	5-6
Suppl. Figure S05: MALDI MSI single pixel spectra.	7
Suppl. Figure S06: H&E images.	8
Suppl. Table 01: Precursor ions of detected phosphatidylglycerols in liver and brain.	9

Supplementary Methods

Lipid Extraction and DPPG quantification. After preparation of serial 10 µm sections from liver and brain for MALDI-MSI a serial thick sections of approx. 100 mg wet weight were taken with a scalpel and homogenized with an Ultra Turrax T25 (IKA, Staufen, Germany) in 900 µl PBS. Protein content of homogenates were determined with a Pierce™ BCA Protein Assay Kit (Thermo Scientific, Dreieich, Germany). For DPPG quantification In-Vial Dual Extraction for direct LC MS analysis[33] was used. Therefore 10 µl of homogenates were transferred into MS vial and 50 µl PBS was added. For protein precipitation 30 µl water and 120 µl methanol were added and the vial was vortexed for 2 minutes. To each sample 600 µl MTBE including internal standard (50 pmol/sample PG(16:0;D_{31}-18:1); Avanti Polar Lipids) was included and vials were shaken for one hour at room temperature. Finally 150 µl water was added and samples were centrifuged for 10 min at 3,000 g to achieve complete phase separation. 10 µl of the organic phase were injected from each sample for DPPG quantification by MS analysis. UPLC-ESI-(QqQ)MS^2 analysis was performed with a Xevo TQ-S tandem mass spectrometer instrument (Waters GmbH, Eschborn, Germany) coupled to an automated Acquity I-Class UPLC using an Acquity UPLC® CSH C18 column 1.7 µm (2.1x100 mm) (Waters GmbH, Eschborn, Germany). Separation was carried out following a 13 min multistep linear gradient. From 0 to 0.4 min solvent B was increased from 43% to 50%, from 0.4 to 4 min B was increased to 70%, then to 95% in 6 min, to 99% in 1 min, then 99% B was maintained for 1 min and decreased to 43% B in 1 min and then maintained for another 1 min for column re-equilibration. The flow-rate was 0.35 ml/min, mobile phase A consisted of methanol in water (1/1, (v/v)), ammonium formate (10 mM), formic acid (0.1%), citrate (5 µM) and B consisted of *iso*-propanol/methanol (99/1, (v/v)), ammonium formate (10 mM), formic acid (0.1%), citrate (5 µM). MassLynx™ (v 4.1 SCN 843) software version (Waters GmbH, Eschborn, Germany) was used for instrument control and data acquisition. Phosphatidylglycerols including DPPG were detected in multiple reaction monitoring (MRM) mode scanning for both, the protonated and the ammoniated molecular ions (first transition, $[M+H]^+ \rightarrow [M+H-172]^+$ and second transition, $[M+NH4]^+ \rightarrow [M+NH4-189]^+$ (Suppl. Table 01). The intensities of both transitions were added up for quantification. The capillary voltage was set at 2.7 kV, whereas the cone and the source offset were fixed at 50 V. The source and desolvation temperatures were maintained at 90 and 250°C, respectively. The desolvation gas was delivered at 800 l/h, while the cone gas and the collision gas flow were fixed at 150 l/h and 0.15 ml/min, respectively. DPPG was detected at optimized collision energy at 13 eV. The dwell time was adjusted automatically to the number of SRM runs per gradient and was between 4 and 10 ms.

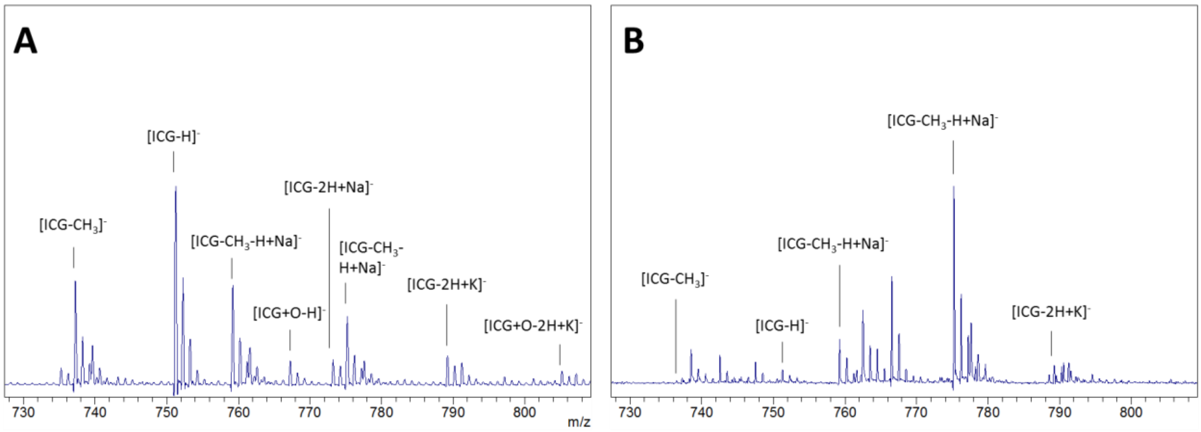

Suppl. Figure S01: MALDI spectra of ICG. MALDI spectra of 5 pmol/µL ICG **(A)** in acetone on a steel target and **(B)** spotted onto liver tissue slices (10 µm). Spectra were measured with PhCCAA matrix (5 mg/mL in acetone/water (9:1, v/v)). Matrix solution was either pipetted onto ICG/acetone after air-drying of the analyte on a steel target (A) or sprayed on tissue slices (B).

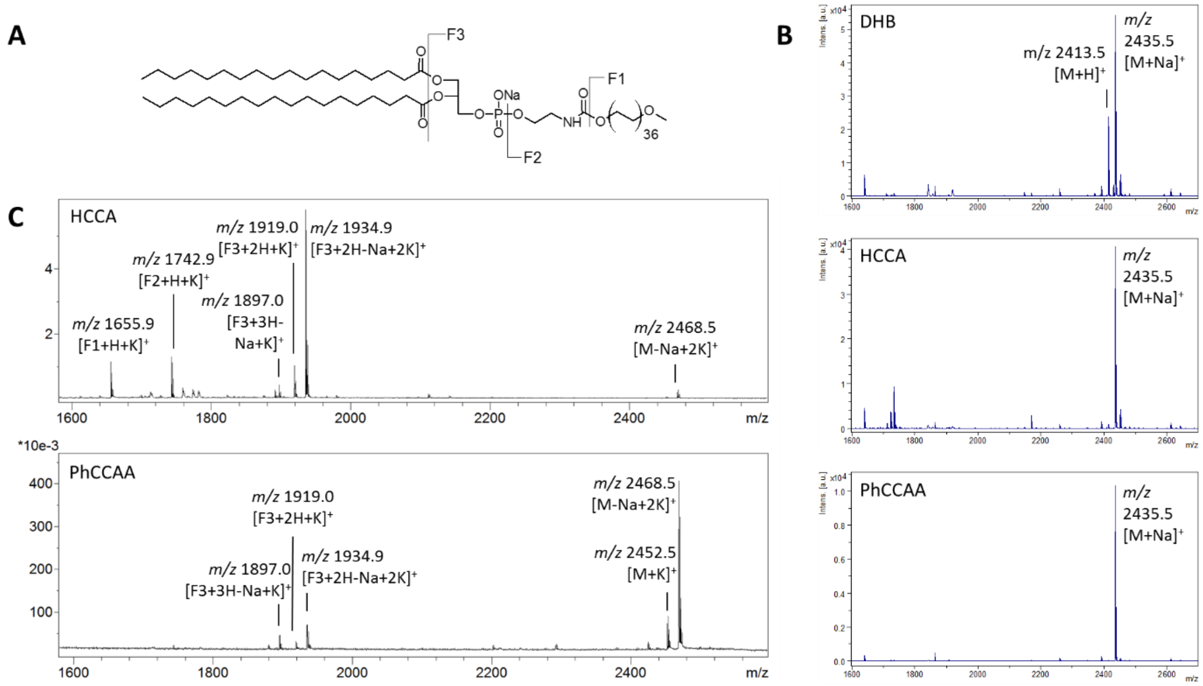

Suppl. Figure S02: Structure of PEG$_{36}$-DSPE and MALDI-TOF spectra. (A) Structure of PEG$_{36}$-DSPE and expected prominent fragments. **(B)** MALDI-TOF spectra of 0.5 mg/mL PEG$_{36}$-DSPE on a steel target measured with DHB matrix (60 mg/mL in acetone/water (90:10, v/v)), HCCA matrix (5 mg/mL in acetone/water (90:10, v/v)) or PhCCAA matrix (5 mg/mL in acetone/water (90:10, v/v) in reflector positive mode. **(C)** MALDI-TOF spectra of PEG$_{36}$-DSPE in liposomes in mouse liver tissue after liposomal administration measured with HCCA[18] or PhCCAA matrix (5 mg/mL in acetone/water (90:10, v/v)) in reflector positive mode.

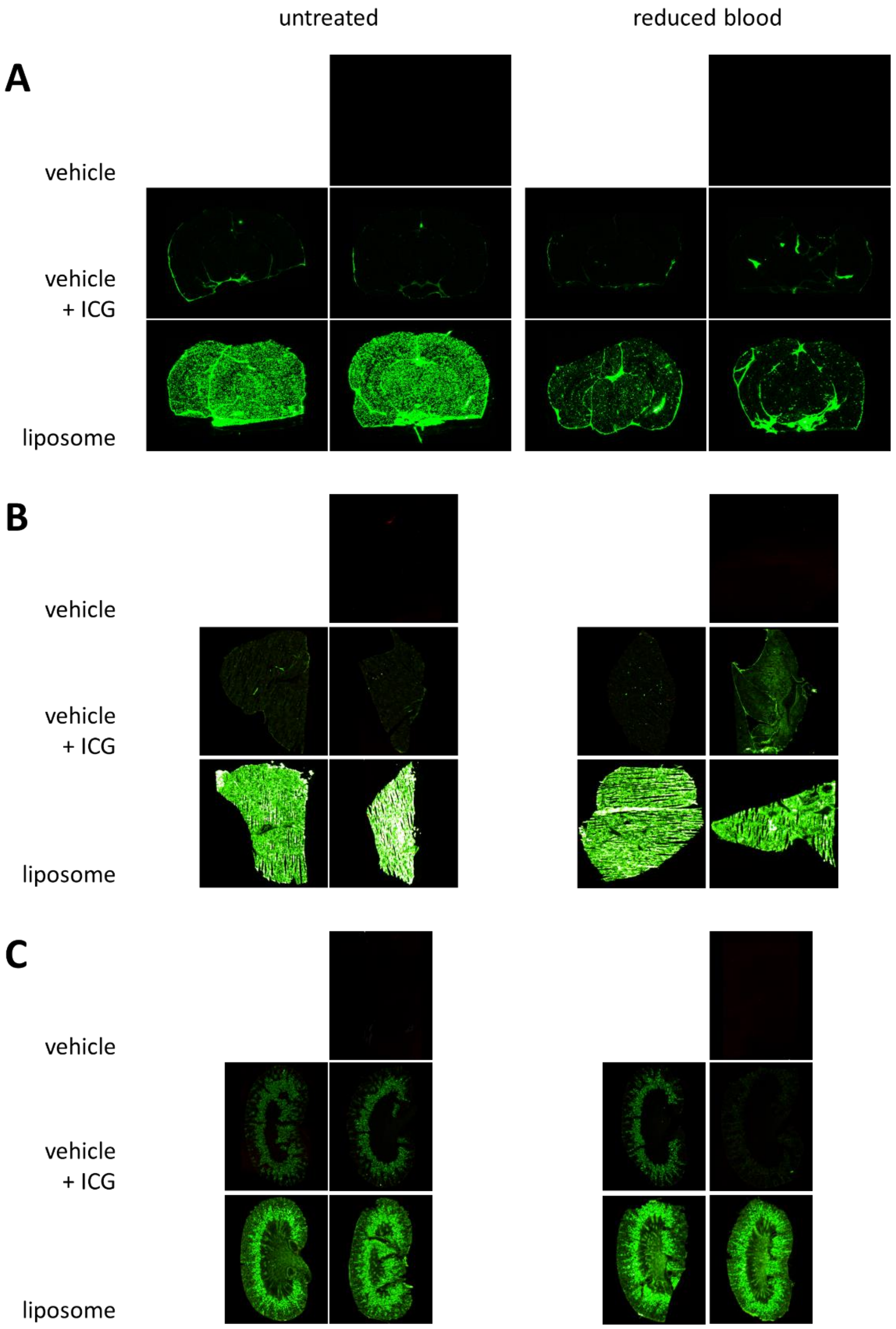

Suppl. Figure S03: Fluorescence images. Fluorescence images of ICG performed on 10 µm slices of **(A)** brain, **(B)** liver or **(C)** kidney of mice that were either dosed with vehicle (PBS) (upper panel), ICG + vehicle (ICG in PBS) (middle panel) or liposomes containing DPPG, PEG_{36}-DSPE and ICG (lower panel). Half of the animals in each group were sacrificed-perfused to eliminate blood from brain vessels (right side). Images were acquired with a LiCor Odyssey Scanner (21 µm Resolution, 0 mm Offset) in the highest quality. Channel sensitivity was optimized for the complete set of tissue sections. Sensitivity was set to 3.5 (brain) or 1.5 (kidney and liver) for the 800 nm channel and to 1.0 for the 700 nm channel.

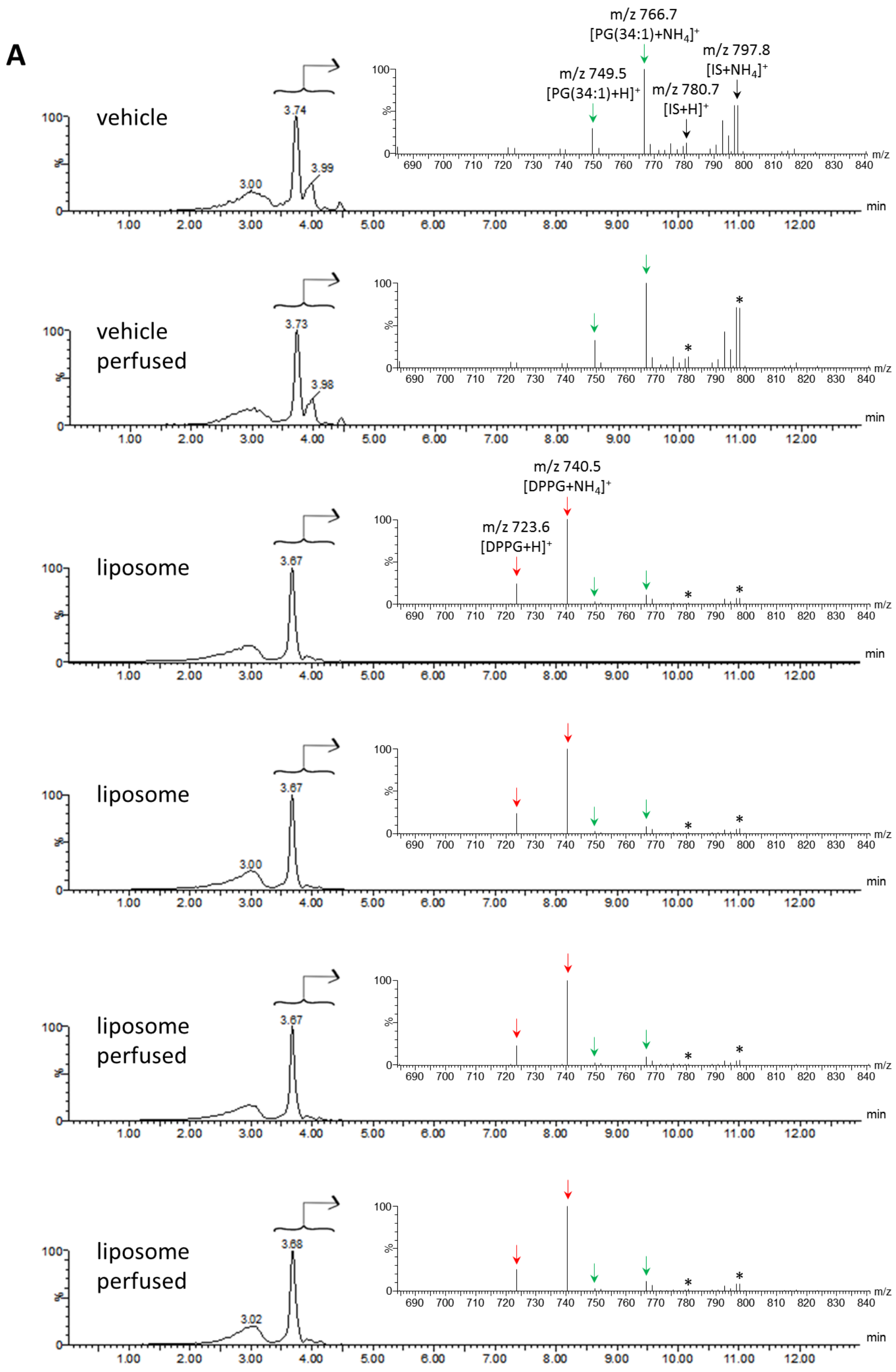
A
m/z 766.7
[PG(34:1)+NH4]+
m/z 797.8
[IS+NH4]+
m/z 749.5
[PG(34:1)+H]+
m/z 780.7
[IS+H]+
vehicle
vehicle
perfused
m/z 740.5
[DPPG+NH4]+
m/z 723.6
[DPPG+H]+
liposome
liposome
liposome
perfused
liposome
perfused
m/z
min

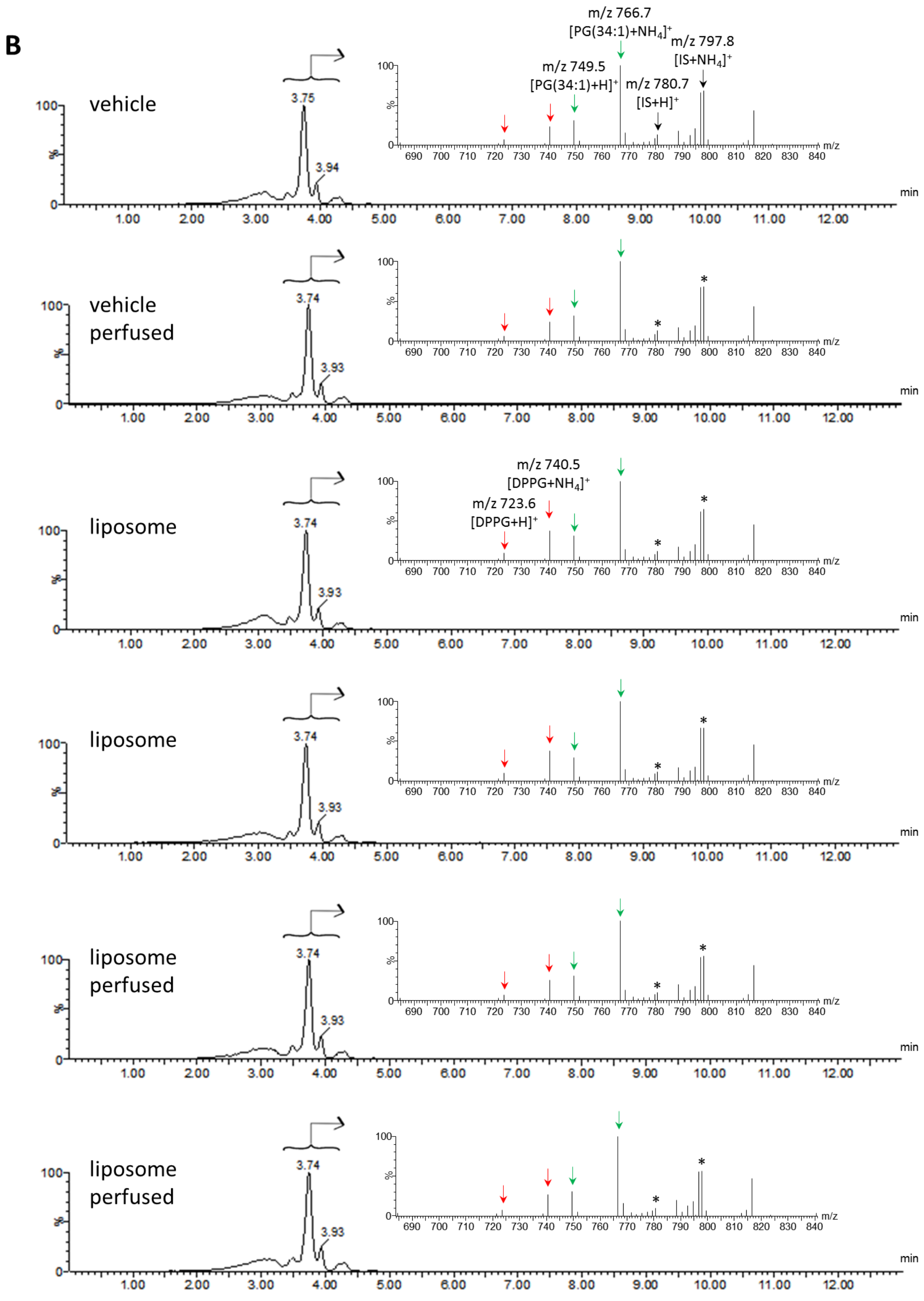

Suppl. Figure 04: UPLC and ESI-(QqQ)MS² profiles of liver and brain extracts. UPLC and ESI-(QqQ)MS² profiles of **(A)** liver and **(B)** brain extracts of six mice that were dosed with vehicle (PBS) or liposomes containing DPPG, PEG_{36}-DSPE and ICG. Bigger spectra represent UPLC-chromatograms of tissue extracts (large plots) and ESI-(QqQ)MS² sum spectra of phosphatidylglycerol species (eluted from 3.4 - 4.6 min; inserts) are presented. Half of the animals in each group were sacrificed-perfused to eliminate blood from brain vessels. Precursor Ions for DPPG are marked with red arrows, whereas precursor ions of the most

prominent phosphatidylglycerol species in liver and brain (PG(34:1)) are marked with green arrows. Signals of the deuterated internal standard (IS) are highlighted with asterisk.

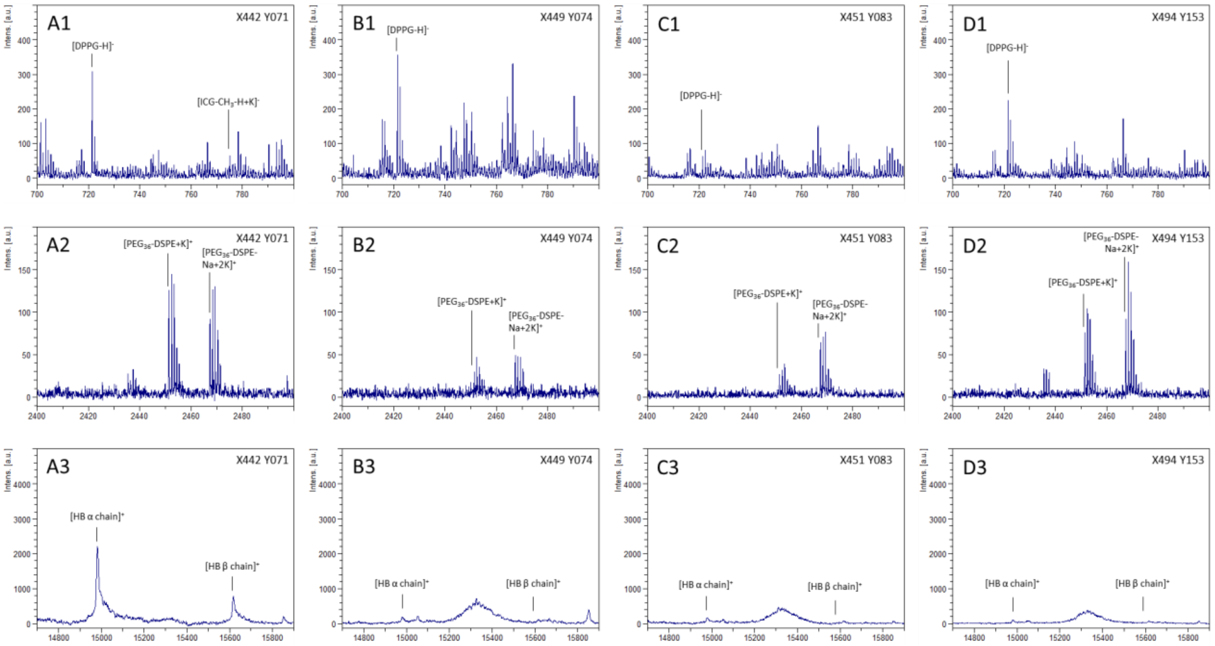

Suppl. Figure 05: MALDI MSI single pixel spectra. MALDI MSI single pixel spectra of motor positions **(A)** X442 Y071, **(B)** X449 Y074, **(C)** X451 Y083, and **(D)** X494 Y153. Spectra of pixels shown in (A) and (B) are illustrated in Figure 05. Liposomal components detected in pixel (A) co-localize with HB and do not enter tissue, while liposomal components detected in pixel (B), (C) and (D) enter brain tissue. Spectra of motor positions (A), (B) and (C) were measured in first mouse brain, while spectra measures on motor position (D) were measured on in a second mouse brain. Spectra of DPPG and ICG in reflector negative ion mode (A1, B1, C1, D1), of PEG_{36}-DSPE in reflector positive ion mode measured with PhCCAA matrix (A2, B2, C2, D2) and spectra of HB in linear positive ion mode measured with sDHB matrix (A3, B3, C3, D3).

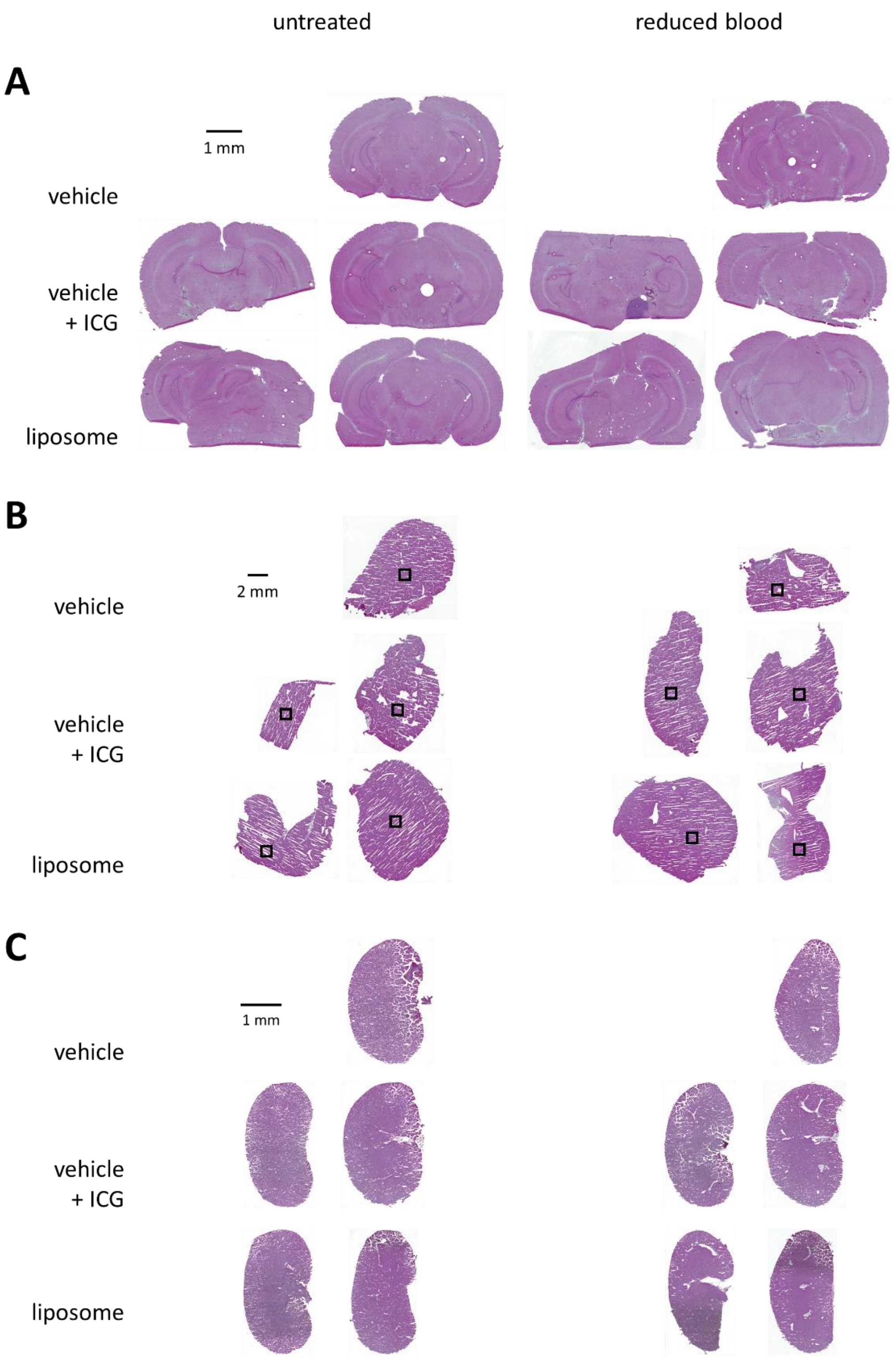

Suppl. Figure S06: H&E images. H&E images on 10 µm slices of **(A)** brain, **(B)** liver or **(C)** kidney of mice that were either dosed with vehicle (PBS) (upper line), ICG + vehicle (ICG in PBS) (middle line) or liposomes containing DPPG, PEG_{36}-DSPE and ICG (lower line). Half of the animals in each group were sacrificed-perfused to eliminate blood from brain vessels (right side). Mapped squares in (B) illustrate the measured region by MALDI MS imaging shown in Figure 02.

Lipid<br>∑FA (C-atoms: double bonds)	Precursor ion<br>$[M+H]^+$<br>[*m/z*]	Precursor ion<br>$[M+NH_4]^+$<br>[*m/z*]	Product Ion<br>[*m/z*]	Collision Energy [V]
PG(32:1)	721.50	738.70	549.49	13
PG(32:0) (DPPG)	723.52	740.50	551.51	
PG(34:1)	749.53	766.70	577.52	
PG(34:0)	751.55	768.70	579.54	
PG(36:4)	771.52	788.54	599.51	
PG(36:3)	773.53	790.56	601.52	
PG(36:2)	775.55	792.70	603.54	
PG(36:1)	777.56	794.70	605.55	
PG(36:0)	779.58	796.70	607.57	
PG(38:6)	795.52	812.54	623.51	
PG(38:5)	797.53	814.56	625.52	
PG(38:4)	799.55	816.58	627.54	
PG(40:6)	823.55	840.58	651.54	
PG(D_{31}-34:1) (IS)	780.72	797.75	608.71	

Suppl. Table 01: Precursor ions of detected phosphatidylglycerols in liver and brain. Phosphatidylglycerols including DPPG were detected in multiple reaction monitoring (MRM) mode scanning for both, the protonated and the ammoniated molecular ions (first transition, $[M+H]^+ \rightarrow [M+H-172]^+$ and second transition, $[M+NH_4]^+ \rightarrow [M+NH_4-189]^+$. The intensities of both transitions were summed up for quantification.
